# Spotlight on the Mucormycosis Outbreak: A Deadly Fungal Infection That Followed the COVID-19 Pandemic

**DOI:** 10.7759/cureus.35095

**Published:** 2023-02-17

**Authors:** Shubham N Patel, Suchi Shah, Jigar Panchal, Chetna Desai, Ila B Upadhya, Masum Patel

**Affiliations:** 1 Medicine, B.J. Medical College, New Civil Hospital Asarwa, Ahmedabad, IND; 2 Pharmacology, B.J. Medical College, New Civil Hospital Asarwa, Ahmedabad, IND; 3 Otolaryngology, B.J. Medical College, New Civil Hospital Asarwa, Ahmedabad, IND

**Keywords:** amphotericin-b, invasive fungal sinusitis, risk-factors, corticosteroid medication, diabetes mellitus, covid 19, rhinocerebral mucormycosis

## Abstract

Background: The COVID-19 pandemic along with its treatment has brought myriad potential complications including the heightened risk of secondary fungal infections like mucormycosis. Mucormycosis is a rare angioinvasive fungal infection that has traditionally been highly fatal despite surgical intervention and antifungal medications.

Aim: To re-evaluate the risk factors, epidemiology, and possible COVID-19-associated conditions on a larger sample size than the existing data.

Methodology: We studied the possible risk factors, clinical presentations, treatment, and outcome of 203 patients with mucormycosis in a single-center retrospective-prospective observational study for three months at a tertiary care hospital after obtaining due permission from the institutional ethics committee.

Results: The mean age of patients was 52 ± 11.5 years, and 92.61% had a history of COVID-19 infection. Around 86.7% of patients were suffering from diabetes mellitus with 50% being already known cases whereas the other 50% developed post-COVID-19 infection; 65.02% of patients were administered corticosteroids during their COVID-19 treatment. About 51.72% of patients required hospital admission and among them, 16.25% of patients required ICU support. The mean oxygen saturation (SpO2) levels on admission were 84.61 ± 12.96%, and 38.92% of patients required mechanical respiratory support. The mean duration between COVID-19 infection and the onset of mucormycosis was 18.80 ± 16.61 days. The most common clinical presentations were facial pain and swelling (26.6%) and ophthalmic symptoms including eye swelling, pain, and ptosis (25.12%). Antifungal treatment was given to all the patients and 89.36% of the patients underwent surgical debridement of fungal mass. At the end of three months, 60.59% of the 188 patients survived with improvement, 13.30% had no improvement and/or deterioration of health, and 18.72% succumbed to mucormycosis. Intracranial involvement and leukocytosis were positively associated with mortality whereas surgical intervention was significant for positive outcomes at the end of three months in patients with mucormycosis (p<0.05).

Conclusion: The sudden rise of mucormycosis during the second wave of COVID-19 can be attributed to uncontrolled blood sugar levels along with high corticosteroid usage as well as various nosocomial factors during the COVID-19 treatment. Early and aggressive treatment with surgical intervention and antifungal drugs can improve disease outcomes.

## Introduction

The COVID-19 or severe acute respiratory syndrome coronavirus 2 (SARS-CoV-2) was declared a global pandemic by the World Health Organisation (WHO) in March 2020 [1]. The pandemic continued to be an ongoing public health concern with more than 275 million cases recorded and more than 5 million deaths globally. In India, more than 34 million cases are reported to date [2]. For some people, opportunistic fungal infections by mucormycosis, candidiasis, and aspergillosis proved to be more morbid than COVID-19 itself [1].

Mucormycosis is an angio-invasive fungal infection caused by the ubiquitous filamentous fungi of the class of Mucoromycetes resulting in vessel thrombosis and tissue necrosis [3]. Mucorales are thermotolerant organisms and are known to infect via ingestion of contaminated food, inhalation of spores, or inoculation into disrupted skin or wounds leading to localized and disseminated infections, especially in an immunocompromised patient [4]. The spectrum of mucormycosis includes rhino-orbital-cerebral, pulmonary, cutaneous, gastrointestinal, and disseminated forms; the most common being rhino-cerebral mucormycosis (ROCM) [5].

The estimated prevalence of mucormycosis in India before COVID-19 was as low as 0.02 to 9.5 cases per 100,000 population but the country witnessed an alarming surge in the number of mucormycosis cases during the COVID-19 pandemic [6]. Spellberg et al. proposed that the viral infection leads to an overexpression of inflammatory cytokines and impaired cell-mediated immunity (cluster of differentiation (CD)4 and CD8 T-cells) which increases the susceptibility to opportunistic fungal infections. Major risk factors for mucormycosis include uncontrolled diabetes mellitus (DM), diabetic ketoacidosis (DKA), metabolic acidosis, malignant hematologic disorders, and deferoxamine therapy in patients receiving hemodialysis [7]. Additionally, factors such as corticosteroids and humidifier use during oxygen therapy in COVID-19 management contributed to the process [8].

Despite aggressive treatment protocols involving surgical intervention and administration of antifungal drugs (amphotericin B, posaconazole, and isavuconazole) mortality rate is increasing in patients of mucormycosis with COVID-19 infection. So, in this study, we aim to assess the risk factors in patients suffering from invasive fungal infection, especially mucormycosis, at a tertiary care teaching hospital.

## Materials and methods

Study design

This is a retrospective-prospective observational, single-center study conducted at the mucormycosis ward of the B.J. Medical College & Civil Hospital, Ahmedabad, Gujarat, India. Due ethics approval was obtained from the Institutional Ethics Committee of the B.J. Medical College & Civil Hospital (approval no. EC/Approval/67/2021/12/06/2021) before starting the study. All patients admitted in this mucormycosis ward in the 15 days of data collection and who met the undermentioned inclusion criteria were included in the study and were followed up for three months.

The inclusion criteria for the study are as follows: patients above the age of 18 years and willing to give written informed consent; patients whose mucormycosis was defined by clinical-radiological diagnosis and confirmed by positive biopsy results before admission to the ward. All the patients who were determined to have COVID-19 are those who tested positive in RT-PCR testing of the throat and nasopharyngeal swabs. Patients who were not willing to give written consent were excluded. The included patients were followed up telephonically at the end of three months for updates on any further surgery, medications, and the clinical outcome. Written informed consent was taken either from the patient or nearest of kin. 

The investigator visited the mucormycosis ward at the Civil Hospital and enrolled the patients as per inclusion criteria and recorded the data in case record forms (CRF). Patient details were recorded during the hospital stay and follow-up details were obtained at the end of three months, telephonically. A total of 203 patients were enrolled in the study.

At the first visit, the following baseline data were collected: demographic details, personal history, past history, COVID-19-related treatment, and clinical history. And during hospitalization, details of ICU stay, oxygen requirement, existing co-morbid conditions, hematological investigations, and treatment data were recorded. At the end of three months, the outcome of mucormycosis was recorded.

Data were entered in Microsoft Excel 2019 (Microsoft Corp., Redmond, WA, USA) worksheet and was analyzed in Excel 2019 itself for various risk factors associated with mucormycosis. Clinical outcome in the patients was analyzed using descriptive statistics. Descriptive statistics were presented as mean and standard deviation (SD) for quantitative variables, and as frequencies with percentages for qualitative data. The chi-square test was used to check the association of risk factors and outcomes in patients with mucormycosis using GraphPad Prism version 9.3.0. (GraphPad Software Inc., San Diego, CA, USA). A p-value <0.05 was considered statistically significant.

## Results

Demographic details

Out of the 203 patients enrolled, 146 (71.92%) were males and 57 (28.07%) were females with a mean age of 52 ± 11.50 years. Around 63.5% of patients belonged to the 40 to 59 years age group. The mean BMI of the patients was 24.43 ± 4.21 kg/m2; 116 patients were healthy, and 49 patients were overweight. Around 37.93% of patients were farmers by occupation followed by housewives at 21.67%. Ninety (44.33%) patients had no addiction history, and 113 (55.67%) patients were found to be addicted to tobacco/alcohol/smoking.

Risk factors

Co-morbidities

Out of 203 patients, 185 (91.13%) were COVID-19-positive and 18 (8.86%) were COVID-19-negative with a mean COVID-19 duration of 14.43 ±5.87 days. The most common comorbidity observed was DM in 176 (86.7%) patients; out of which 88 patients had a history of DM pre-COVID-19 and 88 patients developed DM after the onset of COVID-19. Apart from that, 64 (31.5%) patients had hypertension, 26 (12.8%) patients had DKA, 19 (9.35%) patients had cardiovascular disorders, and 12 (5.91%) patients had respiratory disorders. Other co-morbidities observed were hypothyroidism at 3.44%, history of (H/O) tuberculosis at 2.95%, H/O fungal infection at 2.46%, cancer at 1%, and H/O transplantation at 0.5%.

COVID-19 and Mucormycosis

Out of 203 patients, 162 (73.80%) were non-vaccinated and 41 (20.19%) were vaccinated against COVID-19. Mucormycosis was diagnosed in 74 (40%) patients who had active COVID-19 infection, and 111 (60%) patients were diagnosed with mucormycosis post-COVID-19 infection. Based on the high-resolution computed tomography (HRCT) score, 25 (15.82%) patients had mild, 118 (74.68%) patients had moderate, and 15 (9.49%) patients had severe COVID-19 infection. Out of the 185 patients diagnosed with COVID-19, 105 (56.75%) were hospitalized with a mean duration of hospitalization of 11.35 ± 8.22 days. The medications administered during the COVID-19 treatment are in Table 1.

**Table 1:** COVID-19 medication history of patients who suffered from mucormycosis

Medication (n=203)			Patients administered with drugs
Corticosteroids ( IV or Oral )			65.02% (132)
Duration of steroids	<5 Days		3.44% (7)
	5-10 days		34.97% (71)
	>10 days		27.09% (55)
Remdesivir			33.0% (67)
Tocilizumab			7.88% (16)
Favipiravir			56.15% (114)
Zinc supplements			81.2% (165)
Iron supplements			18.22% (37)
Anticoagulants			1.08% (124)
Anti-microbials (n=203)		Azithromycin	83.25% (169)
		Doxycycline	12.80% (26)
		Ivermectin	29.55% (60)

The mean SpO2 levels on admission (n=157) were 84.61 ± 12.96 (Spo2: 40.12% of patients had >90; 59.87% of patients had <=90), and 80 (43.24%) patients were given home treatment. Intensive care unit treatment was required in 33 (31.43%) hospitalized patients and the mean ICU stay duration was 6.9 ± 6.17 days. Around 79 (75.2%) patients required respiratory support (12.6% of patients required mechanical ventilation), and 26 (24.8%) patients did not need respiratory support. The mean duration of mechanical ventilation was 8.7 ± 8 days. Forty-three patients used only a single mask (20 nasal cannulas, 18 simple oxygen masks, and five non-rebreather masks (NRBM)) for the whole duration of treatment whereas 36 patients used multiple masks for ventilation. The mean duration from the onset of COVID-19 to mucor symptom presentation was 18.80 ± 16.61 days. The mean time between mucormycosis symptoms appearance and treatment was 10.86 ± 10.47 days.

Clinical presentation

**Figure 1:**
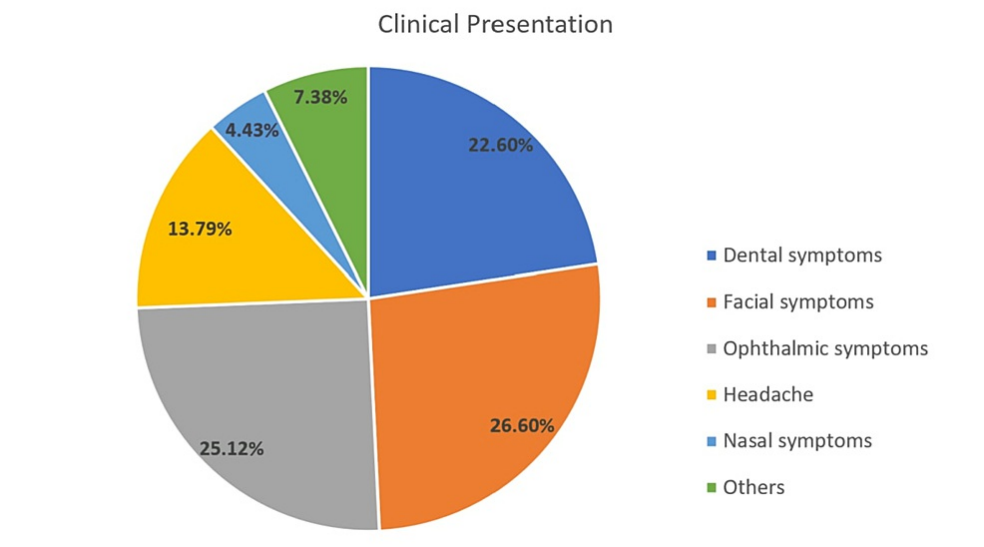
Clinical presentation of mucormycosis

The most common clinical presentations were facial pain and swelling (26.6%) and ophthalmic symptoms including eye swelling, pain, and ptosis (25.12%). Dental pain and moving teeth (22.6%), headache (13.79%), and nasal symptoms (4.43%) were also observed. Other symptoms were paralysis, fever, weakness, and unconsciousness (7.38%). The presentations are demonstrated in Figure 1.

Medical management of mucormycosis

All the patients in our study were administered intravenous amphotericin. Patients were started on amphotericin B or liposomal form in some cases as soon as admission and were shifted to either liposomal or lipid complex preparations in case of hazardous effects on the other systems. The patients after discharge were prescribed oral posaconazole tablets as continuation therapy. Table 2 states the drugs administered to patients and the doses of amphotericin administered. Two patients each opted for ayurvedic and homeopathic therapies.

**Table 2:** Drugs administered during mucormycosis treatment (n=203)

Mucormycosis medication	Value
Conventional amphotericin B	82.27% (167)
Mean doses administered	23.12 ± 10.27
Liposomal amphotericin	38.92% (79)
Lipid complex amphotericin	6.89% (14)
Posaconazole therapy	76.84% (156)
Mean duration of posaconazole (days)	34.19 ± 21.26

Surgical intervention

**Figure 2:**
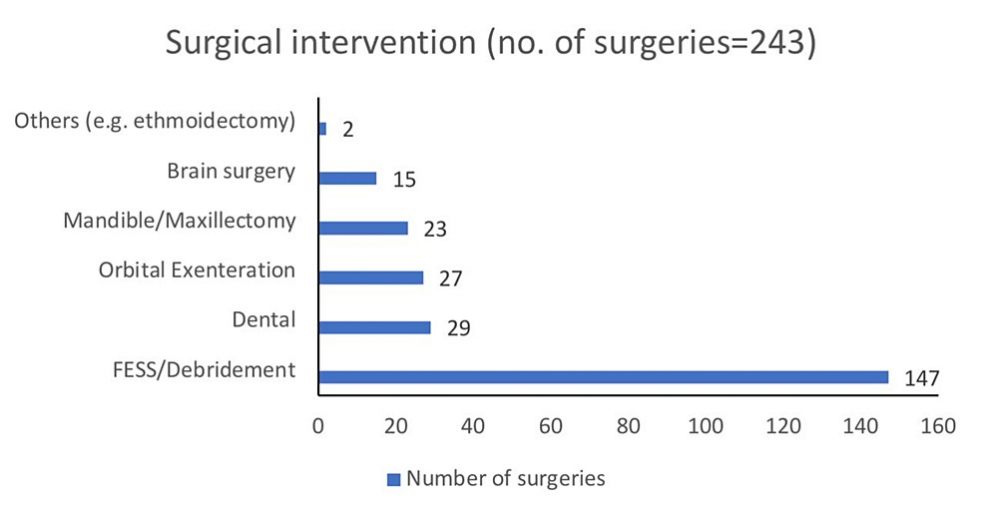
Surgical intervention in patients with mucormycosis FESS: Functional endoscopic sinus surgery

Out of 188 patients, 168 (89.36%) patients underwent surgical removal of the fungal mass whereas 20 patients (10.63%) did not. Sixty-three patients had had revision surgeries (more than one) while 105 had a one-time surgery (total number of surgeries=243). The most common surgery was functional endoscopic sinus surgery (FESS)/debridement in 60.49% of patients followed by dental procedures in 11.93% of patients followed by orbital exenteration in 11.11% of patients. Surgical intervention was found to have a positive impact on the outcome at the end of the follow-up period (p-value=0.034). Figure 2 demonstrates the surgical interventions done during the treatment of mucormycosis.

Outcomes of mucormycosis

**Figure 3:**
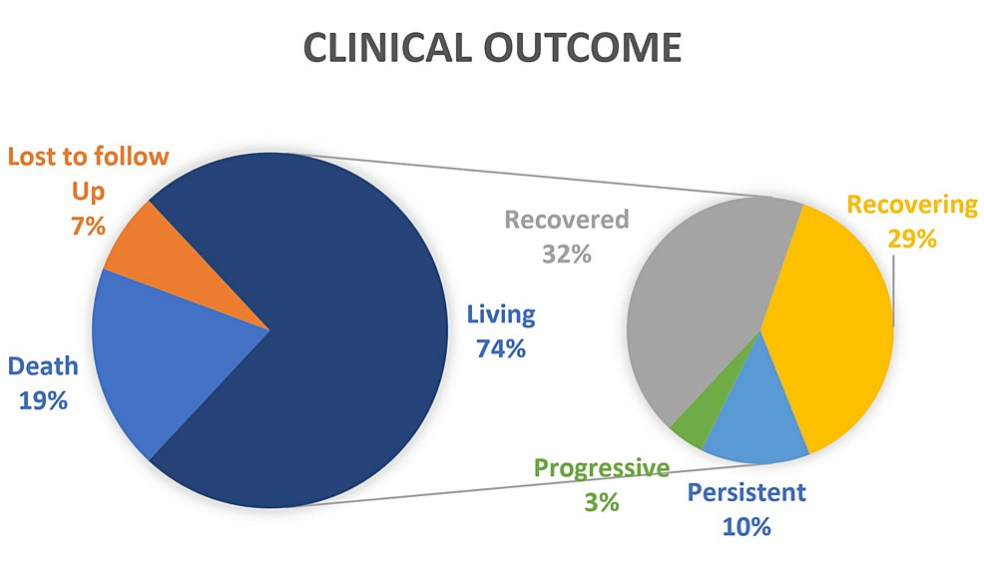
Outcomes of mucormycosis at the end of three months

At the end of three months, 150 patients survived (65 recovered, 58 were recovering, 20 had persistent symptoms, and seven had progressive symptoms), and 38 patients died. Fifteen patients were lost to follow-up. Figure 3 represents the comparison between the clinical outcome at the end of the follow-up period.

Table 3 depicts the statistical significance of various factors in the clinical outcome at the end of the follow-up period and is compared with the statistical significance of those factors determined as risk factors of mucormycosis by a contemporary study [9].

**Table 3:** Association of risk factors with the outcome of mucormycosis at the end of three months HRCT: High-resolution computed tomography, SpO2: Oxygen saturation *p-value <0.05 was considered statistically significant ** The p-value or statistical significance for the risk factors for the occurrence of mucormycosis is adopted from the study by Gupta et al. [9] for the comparison of factors and their roles in the occurrence and outcomes of mucormycosis

Risk factors (n=188)	Survivors	Non-Survivors	p-value	p-value for the occurrence of mucormycosis **
Diabetes mellitus (DM)	129 (79.14)	34 (20.85)	0.86	0.001
Known cases	64	15	0.74	0.035
Newly diagnosed DM	65	19	0.56	0.04
Diabetic ketoacidosis	17 (70.8)	7 (29.2)	0.63	
Hypertension	47 (80)	12 (20)	0.85	0.74
Cardiovascular disorders	12 (75)	4 (25)	0.45	
Respiratory disorders	10 (83)	2 (17)	0.71	
Tuberculosis	4 (80)	1 (20)	1	
Hypothyroidism	5 (83.3)	1 (16.7)	0.71	
History of fungal infection	3 (75)	1 (25)	0.49	
History of acute conditions in the past 3 months	44 (85)	8 (15)	0.27	
COVID-19 vaccination (non-vaccinated)	121 (81.2)	28 (18.8)	0.30	
COVID-19 Positive	138 (74.59)	35 (18.91)	0.45	
Mucor diagnosis during active COVID-19 (n=185)	54 (77.1)	16 (22.9)	0.48	
Spo2 (n=157) =<90	74 (82.2)	16 (17.8)	0.56	
HRCT score	99(79.2)	26(20.8)	0.86	
Intracranial mucor involvement	36 (64.2)	20 (35.8)	0.0006*	
ICU admission	27 (84.3)	5 (15.7)	0.46	
Neutrophil/lymphocyte ratio High (>3.53)	65 (73)	24 (27)	0.08	0.93
Leukocytosis (>10000/cu.mm) [Approximately equal to the average = 10500]	61 (70.1)	26 (29.9)	0.01*	0.56
Eosinophils % (> average eosinophils = 2.83%)	18	88	0.20	<0.0001
Steroid use	98 (79.6)	25 (20.4)	1	0.2
Steroid duration (More than the guideline)	21 (77.7)	6 (2)	0.73	0.001
Remdesivir	51	13	0.98	0.004
Tocilizumab use	12 (75)	4 (25)	0.49	0.55
Zinc	121 (79)	32 (21)	0.58	
Iron	29 (82.8)	6 (17.2)	0.58	
Anticoagulants	90	25	0.51	<0.001
Surgical intervention	141	27	0.034*	

## Discussion

Table 4 contains the comparison of similar parameters in our study with other contemporary studies by Mishra et al. and Selarka et al. [5,1].

**Table 4:** Comparison of the risk factors in our study with two contemporary studies

Parameter			Our study (n=203)	Mishra et al. [5] (n=32)	Selarka et al. [1] (n=47)
Age (mean Age)			52 ± 11.50	58.28 ± 8.57	55 ± 12.80
Diabetes mellitus			86.7%	87.5%	76.6%
Hypertension			31.5%	50.0%	57.4%
Covid severity		Severe	9.49%	56.2%	10.6%
		Non-severe	91.51%	43.8%	89.6%
Corticosteroid usage			65.02%	93.7%	95.7%
Remdicivir usage			33.0%	-	74.4%
Features	Headache		13.79%	93.8%	74.5%
	Orbital symptoms		25.12%	59.4%	40.4%
	Facial symptoms		26.60%	-	34.0%
	Nasal symptoms		4.43%	62.5%	-
Surgical intervention			89.36%	93.8%	80.9%
Total leukocyte count			10104 ± 4413	11478 ± 3693	-
Timelag (COVID-19 and mucormycosis)			18.80 ± 16.61	17.28 ± 11.36	
Mechanical ventilation			38.91%	-	80.85%

Mucormycosis is a rare and potentially fatal invasive fungal infection with an increased risk of occurrence in immunocompromised patients and thus COVID-19 patients are at a higher risk of getting infected [1]. The fungal elements invade the blood vessels leading to pro-coagulative complications like thrombosis, ischaemic infarction, and ultimately necrosis of the host tissue [10]. India has the highest prevalence of mucormycosis around the world, accounting for about 70 times more than the global data [6,11]. A recent study of coronavirus disease-associated mucormycosis in India reported the incidence of mucormycosis to be 0.27% among hospitalized patients and that the caseload had increased about 2.1-fold as compared to the previous year [12]. Mucorales spores invade the body through either inhalation (most common) or ingestion through the mouth. The inhaled spores reach and damage the paranasal sinuses causing necrosis of the tissue structures around and subsequently spreading to the surrounding areas causing local symptoms of invasion and necrosis in case of non-treatment or high fungal load [7]. The majority of the patients in our study were farmers (37.93%) by occupation creating a chance of association with the high exposure of disseminated Mucorales spores in agricultural areas and thus being more susceptible to acquiring the invasive fungal infection.

Diabetes mellitus is the most common risk factor for the development of mucormycosis and is being reported in more than 50% of mucormycosis cases in India [13]. Existing history of diabetes and hyperglycemia was present in 43.34% of our subjects and another 43.43% reported a history of hyperglycemia post the COVID-19 treatment. Post-COVID-19 hyperglycemia was suspected to be associated with the administration of corticosteroids (oral and intravenous) during the treatment of COVID-19. Out of 65.02% of patients who were administered corticosteroids, 16.67% of patients received steroids more than the recommended dose for COVID-19 treatment [14]. Unmonitored use of corticosteroids in COVID-19 treatment and DM leads to an increased susceptibility to contracting mucormycosis due to an interplay of factors causing immunosuppression and hyperglycemia through (I) defective phagocytic mechanism; (II) upregulation of GRP78 receptors in humans and CotH (Mucorales specific protein) in the fungi (entry into human endothelial cells by fungus is facilitated by the interaction of GRP78 and CotH); (III) hyperglycation of iron sequestering proteins causing increased iron delivery to the Mucorales that is essential for their growth [1,15,16].

Hypertension is not an established risk factor for the occurrence and the outcome of mucormycosis but this needs to be further evaluated as there was a significant proportion of subjects with hypertensive history in both our study as well as contemporary studies. Diabetic ketoacidosis is an established risk factor for the occurrence of mucormycosis. The presence of a special ketone reductase system in fungi leads to increased glucose utilization in an acidic medium which boosts fungal growth along with a compromised phagocytic mechanism. In our study, 12.80% of patients had a DKA episode which was higher than in other similar studies where no such cases were reported [5]. History of acute medical conditions (within the past three months before mucormycosis) was present in 26.10% of our patients and could be further investigated as possible risk factors for the development of mucormycosis. These included dental procedures (33.96%), wounds and mucosal exposures (30.18%), upper respiratory tract infections (22.64%), and ophthalmologic procedures (16.98%). The potential modes of transmission of the spores of Mucorales also coincide with the occurrence of these acute medical histories further strengthening the possibility of an association.

The established incubation period of mucormycosis is around seven to 10 days after exposure to the fungus whereas the mean duration between COVID-19 infection and the appearance of mucormycosis symptoms in our study was 18.80 ± 16.61 days. Thus, increasing the possibility of contracting a mucor infection during COVID-19 treatment and hospitalization [17]. Around 31.43% of patients required ICU admission with a mean duration of 6.9 ± 6.17 days where the ICU is already a hotspot for acquiring a multitude of nosocomial and opportunistic infections. Mechanical ventilation was required by 79 (75.2%) out of 105 patients hospitalized for COVID-19 pneumonia for a mean duration of 9.34 ± 8.80 days. Out of 79 patients, 43 patients used only a single mask (20 nasal cannulas, 18 simple oxygen masks, and five NRBM) for the whole duration of treatment whereas the rest of the 36 patients used multiple masks for ventilation. Non-adherence to regular cleaning and replacement of mask may lead to the growth of multiple microorganisms including fungi predisposing the patients to develop mucormycosis. External oxygen in mechanical ventilation is passed through a humidifier which increases the moisture content in the mask, tubing as well as the pharyngeal area of the patient making the conditions favorable for fungal growth. In cases of normal undistilled water usage or non-replacement of the water regularly in the humidifier, a lot of minerals present in tap water along with the moisture play a perfect breeding ground for fungal growth. The oxygen for 60 out of 79 (75.95%) patients was supplied through cylinders whereas only 19 (24.05%) received oxygen produced from in-house oxygen plants. The second wave of COVID-19 was a time when there was a severe shortage of all medical facilities and equipment including oxygen cylinders, so various industrial oxygen suppliers were brought in to fulfill the medical needs. Industrial cylinders are not sterilized before re-filling, and they may have been a source of nutrients and minerals responsible for fungal growth. However, due to the limitations of our study, we could not test the cylinders used but the relation between oxygen delivery systems and the growth of micro-organisms needs to be further studied.

Tocilizumab, an interleukin (IL)-6 inhibitor, is used to treat cytokine storms in COVID-19 patients and was administered in 7.88% of subjects in our study. Tocilizumab acts as an immunomodulator by interleukin suppression and thus may contribute to the occurrence of mucormycosis by downregulation of the natural immune system. Around 81.28% of patients took supplemental zinc during their COVID-19 treatment, which has been demonstrated to increase the fungal growth in vitro due to more efficient substrate utilization and can be one of the factors in the rapid rise of mucormycosis cases among patients being treated for COVID-19 [18]. Around 18.22% of patients had taken supplemental iron therapy which enhances the expression of GRP78 receptors, facilitating fungal entry in cells [16].

The high leucocyte count in 42.70% of patients was associated with unfavorable outcomes at the end of three months and proved to be statistically significant (p-value 0.01). The neutrophil-lymphocyte ratio in our study was high (>3.53) in 49.18% of patients suggestive of relative lymphocytopenia. Lymphocytopenia has been associated with COVID-19 and its severity but its correlation with the occurrence of mucormycosis and outcome needs to be further evaluated [19]. Rhino-cerebral mucormycosis had shown a significant association with a three-month survival rate (p=0.0006) in patients with mucormycosis. 

A delay in diagnosis is an important determinant of the outcome of treatment and even a delay of six days has been seen to almost double the mortality rate [20]. In our study, the mean delay or lag in receiving treatment was 10.86 ± 10.47 days. The lag period in accessing treatment has not been compared with the outcome in this study as the clinical outcome depends on a multitude of factors rather than just delayed diagnosis. The treatment modality of mucormycosis consists of surgical removal of the fungal mass along with antifungal medications like amphotericin B, and posaconazole. In our study, 168 patients underwent surgical removal whereas 20 patients did not. Surgical intervention was a statistically significant parameter positively affecting the outcome at the end of 12 weeks suggesting that aggressive removal or debridement of the invasive fungal mass is a potent step to acutely reduce the progression and in turn obtain a better prognosis.

At the end of 12 weeks, 150 patients survived (32.01% recovered, 28.57% were recovering, 9.85% had persistent symptoms, and 3.44% had progressive symptoms) whereas 18.71% of patients succumbed, and 7.38% of patients were lost to follow-up. Traditionally the survival rates for mucormycosis are low (56% in previous studies), whereas in our study 81% of the patients survived at the end of three months which is attributed to aggressive anti-fungal treatment and early surgical management of patients [21]. The results of our study can be generalized in the aspect of treatment of mucormycosis as we had a good enough sample size from a single center for such a rare disease.

Limitations

Despite having a larger sample size for a single-center study than any of those previously conducted, we did not find significant patients for rare risk factors like transplant, malignancy, etc. Also, being an observational study, we did not have controls that were COVID-19-positive but did not develop mucormycosis to analyze the specific factors that led to the development of the fungal infections. However, to the best of our knowledge and considering the rare occurrence of the disease, this is the largest single-center case series studied.

## Conclusions

The sudden rise of mucormycosis during the second wave of COVID-19 can be attributed to uncontrolled blood sugar levels along with irrational corticosteroid usage combined with various nosocomial factors such as mechanical ventilation and the oxygen used, drug therapy, and ICU admissions during COVID-19 treatment. The role of COVID-19 infection by itself in the causation of invasive fungal infection needs to be evaluated further. Early and aggressive treatment with surgical intervention and antifungal drugs can be pivotal in improving disease outcomes in patients with mucormycosis.

## References

[1] Selarka L, Sharma S, Saini D (2021). Mucormycosis and COVID-19: an epidemic within a pandemic in India. Mycoses.

[2] (2022). India: WHO Coronavirus Disease (COVID-19) Dashboard With Vaccination Data | WHO Coronavirus (COVID-19) Dashboard With Vaccination Data. https://covid19.who.int/region/searo/country/in.

[3] Bala K, Chander J, Handa U, Punia RS, Attri AK (2015). A prospective study of mucormycosis in north India: experience from a tertiary care hospital. Med Mycol.

[4] Reid G, Lynch JP 3rd, Fishbein MC, Clark NM (2020). Mucormycosis. Semin Respir Crit Care Med.

[5] Mishra Y, Prashar M, Sharma D, Akash Akash, Kumar VP, Tilak TV (2021). Diabetes, COVID 19 and mucormycosis: clinical spectrum and outcome in a tertiary care medical center in Western India. Diabetes Metab Syndr.

[6] Prakash H, Chakrabarti A (2019). Global epidemiology of mucormycosis. J Fungi (Basel).

[7] Spellberg B, Edwards J Jr, Ibrahim A (2005). Novel perspectives on mucormycosis: pathophysiology, presentation, and management. Clin Microbiol Rev.

[8] Yang X, Yu Y, Xu J (2020). Clinical course and outcomes of critically ill patients with SARS-CoV-2 pneumonia in Wuhan, China: a single-centered, retrospective, observational study. Lancet Respir Med.

[9] Gupta D, Kulkarni R, Pujari S (2021). Covid-19 associated mucormycosis: a case-control study. MedRxiv.

[10] Afroze SN, Korlepara R, Rao GV, Madala J (2017). Mucormycosis in a diabetic patient: a case report with an insight into its pathophysiology. Contemp Clin Dent.

[11] Chander J, Kaur M, Singla N (2018). Mucormycosis: battle with the deadly enemy over a five-year period in India. J Fungi (Basel).

[12] Patel A, Agarwal R, Rudramurthy SM (2021). Multicenter epidemiologic study of coronavirus disease-associated mucormycosis, India. Emerg Infect Dis.

[13] Prakash H, Ghosh AK, Rudramurthy SM (2019). A prospective multicenter study on mucormycosis in India: Epidemiology, diagnosis, and treatment. Med Mycol.

[14] (2022). Clinical management protocol for covid-19 (In Adults) Ministry of Health and Family Welfare, Government of India. https://www.mohfw.gov.in/pdf/UpdatedDetailedClinicalManagementProtocolforCOVID19adultsdated24052021.pdf.

[15] Ibrahim AS, Spellberg B, Walsh TJ, Kontoyiannis DP (2012). Pathogenesis of mucormycosis. Clin Infect Dis.

[16] Alqarihi A, Gebremariam T, Gu Y (2020). GRP78 and integrins play different roles in host cell invasion during mucormycosis. mBio.

[17] Smith RM, Lee J, Mody RK (20151). Determining the incubation time of mucormycosis: a systematic review. Open Forum Infect Dis.

[18] Wegener WS, Romano AH (1963). Zinc stimulation of RNA and protein synthesis in rhizopus nigricans. Science.

[19] Guan WJ, Ni ZY, Hu Y (2020). Clinical characteristics of coronavirus disease 2019 in China. N Engl J Med.

[20] Chamilos G, Lewis RE, Kontoyiannis DP (2008). Delaying amphotericin B-based frontline therapy significantly increases mortality among patients with hematologic malignancy who have zygomycosis. Clin Infect Dis.

[21] Jeong W, Keighley C, Wolfe R, Lee WL, Slavin MA, Kong DC, Chen SC (2019). The epidemiology and clinical manifestations of mucormycosis: a systematic review and meta-analysis of case reports. Clin Microbiol Infect.

